# Efficacy of orthodontic mini implants for en masse retraction in the maxilla: a systematic review and meta-analysis

**DOI:** 10.1186/s40729-018-0144-4

**Published:** 2018-10-25

**Authors:** Kathrin Becker, Annika Pliska, Caroline Busch, Benedict Wilmes, Michael Wolf, Dieter Drescher

**Affiliations:** 1 Department of Orthodontics, Universitätsklinikum Düsseldorf, 40225 Düsseldorf, Germany; 2 Department of Orthodontics, Universitätsklinikum RWTH Aachen, Aachen, Germany

**Keywords:** Bone screws, Orthodontic anchorage procedures, TAD, En masse retraction, Mini implants, Micro implants, Systematic review, Meta-analysis

## Abstract

**Background/aim:**

Retraction of the upper incisors/canines requires maximum anchorage. The aim of the present study was to analyze the efficacy of mini implants in comparison to conventional devices in patients with need for en masse retraction of the front teeth in the upper jaw.

**Material and methods:**

An electronic search of PubMed, Web of Science, and EMBASE and hand searching were performed. Relevant articles were assessed, and data were extracted for statistical analysis. A random effects model, weighted mean differences (WMD), and 95% confidence intervals (CI) were computed for horizontal and vertical anchorage loss at the first molars in the analyzed patient treatments.

**Results:**

A total of seven RCTs employing direct anchorage through implants in the alveolar ridge were finally considered for qualitative and quantitative analysis, and further five publications were considered for the qualitative analysis only (three studies: indirect anchorage through implant in the mid-palate, two studies: direct/indirect anchorage in the alveolar ridge). In the control groups, anchorage was achieved through transpalatal arches, headgear, Nance buttons, intrusion arches, and differential moments.

WMD [95% CI, *p*] in anchorage loss between test and control groups amounted to − 2.79 mm [− 3.56 to − 2.03 mm, *p* < 0.001] in the horizontal and − 1.76 mm [− 2.56 to − 0.97, *p* < 0.001] favoring skeletal anchorage over control measures. The qualitative analysis revealed that minor anchorage loss can be associated with indirect anchorage, whereas anchorage gain was commonly associated with direct anchorage. Implant failures were comparable for both anchorage modalities (direct 9.9%, indirect 8.6%).

**Conclusion:**

Within its limitations, the meta-analysis revealed that maximum anchorage en masse retraction can be achieved by orthodontic mini implants and direct anchorage; however, the ideal implant location (palate versus alveolar ridge) and the beneficial effect of direct over indirect anchorage needs to be further evaluated.

**Electronic supplementary material:**

The online version of this article (10.1186/s40729-018-0144-4) contains supplementary material, which is available to authorized users.

## Review

### Background

Extraction of the permanent teeth for retraction of the protruded front teeth is a routine approach in orthodontics. Various techniques such as headgear, Nance button, and transpalatal arches (TPA) have been proposed to achieve sufficient anchorage [5, 8, 9, 12, 28, 31, 45]. Nevertheless, anchorage control turned out to be highly demanding as the conventional approaches were commonly associated with anchorage loss, i.e., mesial migration of the posterior dental anchorage units.

In order to improve anchorage control, differential moments have been described and monitored in clinical studies [25, 26]. The outcomes were promising, but nevertheless, anchorage loss and unexpected space opening most probably due to activation failures have also been reported. Some authors suggested that consecutive canine and front retraction may be more effective than en masse retraction of the front segment to preserve anchorage. Despite this, the effectiveness of this approach is still discussed, and controversial outcomes have been reported [18, 60].

In the past two decades, temporary orthodontic anchorage devices (TADs) including orthodontic mini implants and mini-plates have been introduced to improve anchorage control [39, 56, 57]. Orthodontic implants can be loaded directly after insertion and are usually removed after treatment completion [17, 36]. Therefore, orthodontic mini implants most often have a smooth surface to ease removal [32], whereas mini-plates are more invasive and require surgical intervention and flap preparation [6]. For this reason, orthodontic mini implants are frequently used, and two concepts are predominant: One is to stabilize a dental anchorage unit by connecting it to the implant (indirect anchorage), and the other is to directly load the orthodontic mini implant with the reactive forces (direct anchorage) [42].

Accordingly, there is a need to identify if orthodontic mini implants are more effective to control anchorage compared to conventional devices, and to assess if the direct or indirect anchorage concept is more beneficial. The aim of this systematic review was therefore to address the following question: “In patients with a need for en masse retraction of the upper front teeth, what is the efficacy of orthodontic mini implants for anchorage quality compared with conventional devices?”

## Methods

This systematic review was structured and conducted according to the preferred reporting items of the PRISMA statement [34].

### Focused question

The focused question serving for literature search was structured according to the PICO (*P*atients, *I*ntervention, *C*ontrol, *O*utcome) format: “In patients with a need for en masse retraction of the upper front teeth, what is the efficacy of orthodontic mini implants for anchorage control compared with conventional devices?” According to the PICO convention, this question has been formulated as follows:

- *Patients*: for which subgroups of patients with a need for en masse retraction of the upper incisors/canines
- *Intervention*: do orthodontic mini implants have a benefit over conventional devices?
- *Control*: compared to forgoing orthodontic mini implants (compared to conventional treatment)
- *Outcome*: with regard to treatment efficacy (anchorage control), treatment duration, potential harms (inflammation, implant loss)

### Search strategy

The PubMed database of the US National Library of Medicine, EMBASE, and the Web of Knowledge of Thomson Reuters were used as electronic databases to perform a systematic search for relevant articles published in the dental literature between 1992 and Dec 31, 2017. Furthermore, the Cochrane Central Register of Controlled Trials (CENTRAL) was searched manually.

A commercially available software program (Endnote X7, Thomson, London, UK) was used for electronic title management. Screening was performed independently by two authors (K.B. and M.W.). Disagreement regarding inclusion during the first and second stage of study selection was resolved by discussion.

The combination of key words (i.e., Medical Subject Headings MeSH) and free text terms included:

- *Search terms PubMed/MEDLINE (including MeSH terms)*
  - (“en-masse retraction” OR “incisor retraction” OR “front retraction” OR “orthodontic gap closure” OR or “orthodontic space closure” OR “extraction therapy” [mh])AND (“mini implants” OR “micro screws” OR “micro implants” OR “skeletal anchorage” OR “palatal implant” OR “skeletal” OR “skeletal anchorage” OR “implant” OR “bone screw” OR “temporary anchorage device” OR “TAD” OR “Bone screws”[mh] OR “intraosseous screw” OR “dental implants”[mh])
  - AND (“anchorage loss” OR “anchorage quality” OR “quality of life” OR “benefit” or “harm” OR “efficacy” OR “side effects” OR “effect” OR “orthodontic anchorage procedures”[mh] OR “treatment outcome”[mh])
- *Search terms EMBASE (including EMTREE terms)*
  - (“en-masse retraction” OR “incisor retraction” OR “front retraction” OR “orthodontic gap closure” OR “orthodontic space closure” OR “extraction therapy” [EMTREE]) AND (“mini implants” OR “micro screws” OR “micro implants” OR “skeletal anchorage” OR “palatal implant” OR “skeletal” OR “skeletal anchorage” OR “implant”[EMTREE] OR “bone screw”[EMTREE] OR “tooth implant”[EMTREE] “temporary anchorage device” OR “TAD” OR “Bone screws” OR “intraosseous screw” OR “dental implants”) AND (“anchorage loss” OR “anchorage quality” or “quality of life” OR “benefit” OR “harm” OR “efficacy” OR “side effects” OR “effect” OR “orthodontic anchorage”[EMTREE] OR “treatment outcome”)

### Hand search

The electronic search was complemented by a hand search of the following journals: *American Journal of Orthodontics and Dentofacial Oorthopedics*, *The Angle Orthodontist*, *European Journal of Orthodontics*, *Journal of Orofacial Orthopedics*, *Orthodontics and Craniofacial Research*, and *Seminars in Orthodontics*.

Finally, the references of all selected full-text articles and related reviews were scanned. If required, the corresponding authors were contacted and requested to provide missing data or information.

### Study selection

During the first stage of study selection, the titles and abstracts were screened and evaluated according to the following inclusion criteria:

1. English language
2. Prospective controlled clinical trials (CCT) (for qualitative synthesis) or randomized controlled clinical trials (RCT) (for qualitative and quantitative synthesis, parallel group designs) in humans comparing mini implant based on conventional anchored treatments
3. Patients: general population (all ethnicities, community dwelling)
4. Measurement of anchorage loss of the first upper molars during retraction

At the second stage of selection, all full-text articles identified during the first stage were acquired. During this procedure, the pre-selected publications were evaluated according to the following exclusion criteria:

1. Patients younger than 12 years
2. No bilateral extraction of one upper premolar per site
3. Inclusion of less than five patients
4. Lack of clinical data on anchorage loss
5. Measurement of anchorage loss not by superimposition of lateral cephalograms or superimposition of study casts
6. Previous orthodontic treatment
7. Treatment in control group not specified
8. Inclusion of diseased patients, e.g., patients with systemic diseases, periodontal disease, and syndromes
9. Other treatment than en masse retraction and mini implants
10. Other sources of skeletal anchorage than orthodontic mini implants or micro implants

### Data extraction and method of analysis

At least two review authors examined the titles and abstracts of the identified studies and reports independently. Reports which were clearly not relevant were excluded, whereas full-text documents were retrieved for all potentially relevant studies and eligibility was assessed according for the criteria defined in advance. Disagreements were resolved by open discussion occasionally arbitrated by an independent assessor (D.D.). A data extraction template was generated including the items’ study design, population, type of implants, number of implants, location of the implants, time points of observation, treatment duration, control intervention, measurement method, and primary and secondary outcomes as well as risk of bias (Additional file 1). Data extraction was performed independently by at least two review authors.

For qualitative and quantitative data analysis, the horizontal and vertical anchorage loss values associated with direct and indirect anchorage against a control measure were defined as primary outcomes. For qualitative data analysis, transversal anchorage loss, treatment duration, and implant failures with direct and indirect anchorage were defined as secondary outcomes.

### Quality assessment of selected studies

A quality assessment of all selected full-text articles was performed according to the Cochrane Collaboration’s tool for assessing risk of bias (low, high, unclear) including the following domains: random sequence generation, allocation concealment, blinding of outcome assessment, incomplete outcome data, selective reporting, and other sources of bias.

Quality assessment was performed in two different phases. In the first phase, quality assessment was conducted independently by at least two authors (A.P., C.B., K.B.) based on the published full-text articles. In the second phase, disagreements were resolved by discussion. A risk of bias table was completed for each included study.

### Dealing with missing data and zero values

When data were not available in the printed report, we calculated the missing information whenever possible (e.g., by subtracting pre- and post en masse retraction values). In cases where a zero variance (0.00 mm) was presented in the summary tables, these values were changed to 0.01 mm to enable meta-analysis. The corresponding authors of the published studies were contacted when needed.

### Data synthesis

Heterogeneity among the clinical trials, meta-analysis (i.e., weighted mean differences and 95% confidence intervals, random effects model to account for potential methodological differences between studies), forest plots, and publication bias (Egger’s regression to quantify the bias captured by funnel plots) were assessed using a software program (Review Manager (RevMan) version 5.2. Copenhagen: The Nordic Cochrane Centre, The Cochrane Collaboration, 2012).

## Results

### Description of studies

#### Study selection

The search for the review was undertaken at December 31, 2017. A total of 2046 potentially relevant titles and abstracts were found during the electronic and manual search (676 after duplicate removal) of which 99 titles were considered relevant for abstract screening. During the first stage of study selection, 58 publications were excluded based on the abstract. For the second phase, the complete full-text articles of the remaining 41 publications were thoroughly evaluated. A total of 29 papers had to be excluded at this stage because they did not comply with the inclusion or exclusion criteria of the present systematic review (Table 1).

**Table 1:** List of excluded studies (with reason)

Reference	Reason for exclusion
Barros et al. (2017) [3]	Anchorage loss at first molar not specified
Borsos et al. (2012) [7]	No en masse retraction (two step canine and front retraction)
Dai et al. (2009) [10]	Chinese language
Durrani et al. (2017) [13]	Anchorage loss a first molar not specified
El-Beialy et al. (2009) [14]	Anchorage loss a first molar not specified
Garfinkle et al. (2008) [16]	Anchorage loss at first molar not specified
Heo et al. (2007) [18]	No mini implants used for anchorage
Herman et al. (2006) [19]	Anchorage loss a first molar not specified
Janson et al. (2013) [22]	Anchorage loss a first molar not specified
Jee et al. (2014) [23]	Use of mini implants and mini-plates
Kuroda et al. (2009) [27]	T0 ceph before leveling (anchorage loss not specified during en masse retraction only)
Liu et al. (2011) [29]	Anchorage loss at first molar not specified
Ma et al. (2015) [30]	Full-text unavailable (requested but no response from authors)
Miyazawa et al. (2010) [33]	Anchorage loss at first molar not specified
Monga et al. (2016) [35]	Retrospective study
Park et al. (2004) [38]	Case report
Park et al. (2007) [40]	Case report
Park et al. (2008) [41]	Retrospective study
Santiago et al. (2009) [43]	No en masse retraction, anchorage loss at first molar not specified
Shi et al. (2008) [44]	Extraction of premolars or molars
Thiruvenkatachari et al. (2006) [46]	Canine retraction only
Turkoz et al. (2011) [47]	No premolars extracted
Upadhyay et al. (2012) [51]	No premolar extraction
Gollner et al. (2009) [17]	No premolar extraction
Wehrbein et al. (1996a) [55]	Case report
Wehrbein et al. (1996b) [56]	Case report
Xu et al. (2008) [59]	Language not meeting inclusion criteria
Xun et al. (2004) [61]	Language not meeting inclusion criteria
Yao et al. (2008) [62]	Retrospective study, mini-plates, and mini implants used

**Fig. 1:**
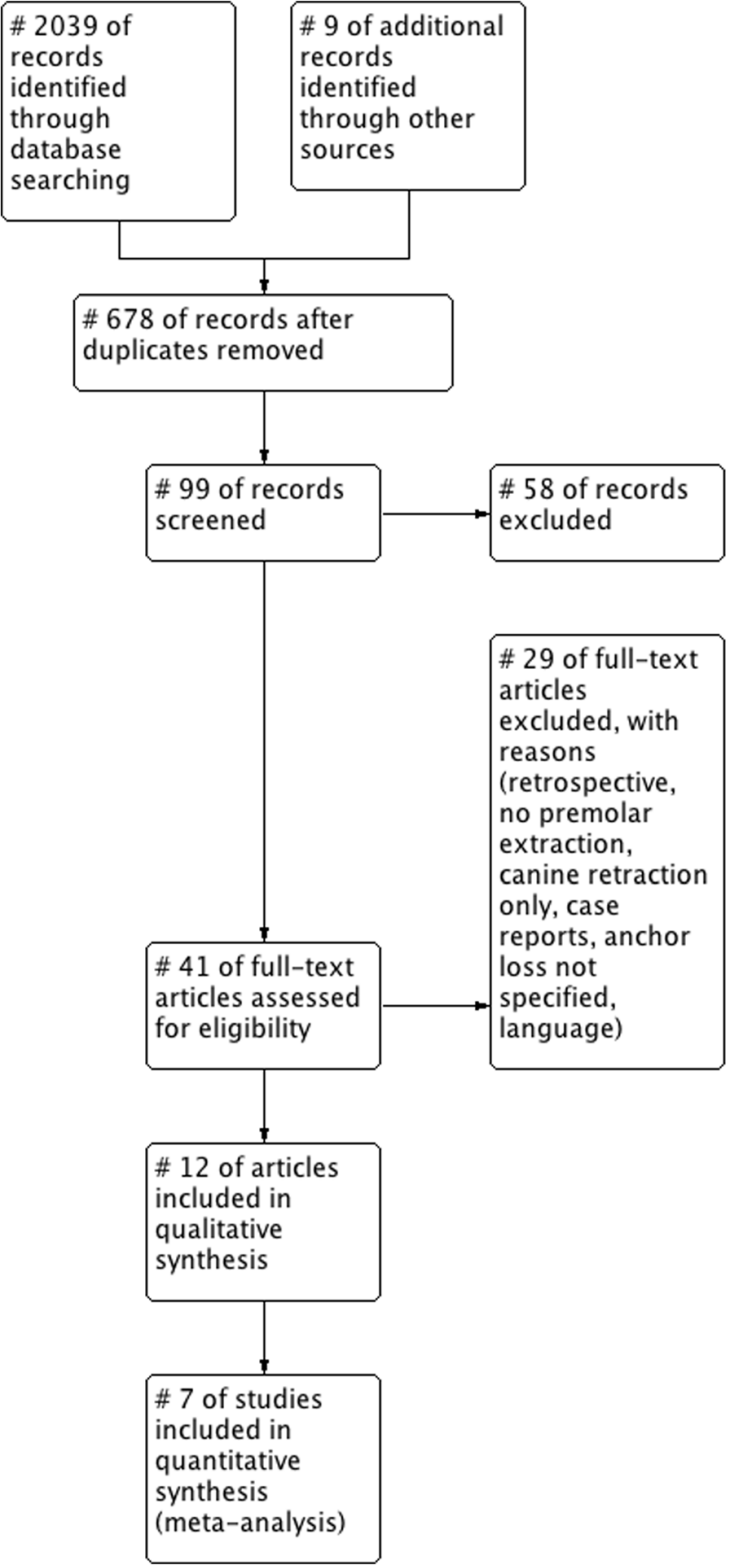
PRISMA study flow diagram

Finally, a total of 12 publications (reporting on 12 studies) were considered for the qualitative and a total of 9 publications for the quantitative assessment (Fig. 1). However, only two RCTs were found comparing indirect anchorage with conventional anchorage devices, whereas 7 studies compared direct anchorage with the control intervention. At this stage, it was decided to perform the quantitative analysis only for the direct anchorage groups. The two studies comparing indirect anchorage with a control intervention were included in the qualitative analysis only. Summary details of the included studies are given in Table 2.

**Table 2:** Characteristics of the included studies (*TPA* transpalatal arch, *RCT* randomized controlled clinical trial, *CCT* controlled clinical trial)

Reference	Number of patients	Type of study (RCT/CCT/other)	Control intervention	Type of implant (length, material)	Number of implants	Location of implant	Mode of anchorage (direct/indirect)
Al-Sibaie and Hajeer [1]	56 (28 implant, 28 non implant)	RCT	TPA	Self-drilling titanium mini implants (1.6 mm diameter and 7 mm length; Tuttlingen, Germany)	2	Between the maxillary second premolar and first molar	Direct
Basha et al. [4]	14 (7 implant, 7 non implant)	RCT	TPA	Surgical steel mini implants (1.3 mm diameter, 8 mm length; SK Surgical, Pune, India.)	2	Placed between the roots of second premolar and first molar in the maxilla	Direct
Benson et al. [5]	51 (23 implant; 24 non implant)	RCT	Headgear	Ortho implant, (6 mm length, Straumann, Waldenburg, Switzerland)	1	Midpalatal	Indirect
Chopra et al. [9]	50 (25 implant; 25 non implant)	RCT	Nance button; lingual arch	Self-drilling titanium ortho implants	4	Buccal alveolar bone between the second premolars and first molars in all the four quadrants	Indirect
Davoody et al. [11]	46 (23 implant, 23 non-implant group)	RCT	Intrusion arch and mushroom loops	1.8–2 mm in width, 8–9 mm in length	4	Placed between maxillary second premolars and first molars in all four quadrants	Direct
Liu et al. [28]	34	RCT	TPA	Self-tapping titanium mini-screw implants (8 mm length, 1.2 mm diameter, Cibei, Ningbo, China)	2	Between the roots of the first molar and the second premolar	Direct
Upadhyay et al. [49]	30 (15 implant, 15 non-implant)	RCT	Treatment in control group not specified: Nance holding arch, extraoral traction, banding of the second molars, and differential moments	Custom made at our institute by modifying conventional surgical screws, measuring 1.3 mm in diameter and 8 mm in length	2	Placed between the maxillary second premolar and first molar, preferably between the attached and movable mucosae	Direct
Upadhyay et al. [48]	23	Other (cohort study)	No control group	Titanium mini implants (1.3 mm in diameter and 8 mm in length)	2	Placed between the roots of the first molar and the second premolar in both upper quadrants	Direct
Upadhyay et al. [50]	40 (20 implant, 20 non implant)	RCT	Conventional methods such as headgears, transpalatal arches, banding of second molars, application of differential moments	Titanium mini implants (1.3 mm diameter, 8 mm length)	4	Between the roots of the first molar and second premolar in all four quadrants	Direct
Victor et al. [52]	20 (10 implant, 10 non-implant)	RCT	NiTi closed coil spring	Absoanchor—SH 1312-08; (1.3 mm diameter, 8 mm length)	4	Placed between the roots of second premolar and first molar in the upper arch, the screw insertion was angulated at 40° and 8 mm gingival to the archwire	Direct
Wehrbein et al. [54]	9	Other (cohort study)	No control group	Orthosystem (diameter 3.3 mm, lengths are 4 and 6 mm)	1	Midpalatal	Indirect
Wilmes et al. [57]	20 (10 in implant group of which 5 patients had additional transversal reinforcement and 5 did not, 10 in non-implant group)	CCT	TPA	2.0 × 10 mm, Dual Top™, Jeil Medical Corporation, Seoul, South Korea, or 2.0 × 11 mm, BENEFIT, Mondeal Medical Systems, Mühlheim a.d. Donau, Germany	1	Placed in the anterior palate	Indirect

#### Risk of bias in the included studies

**Fig. 2:**
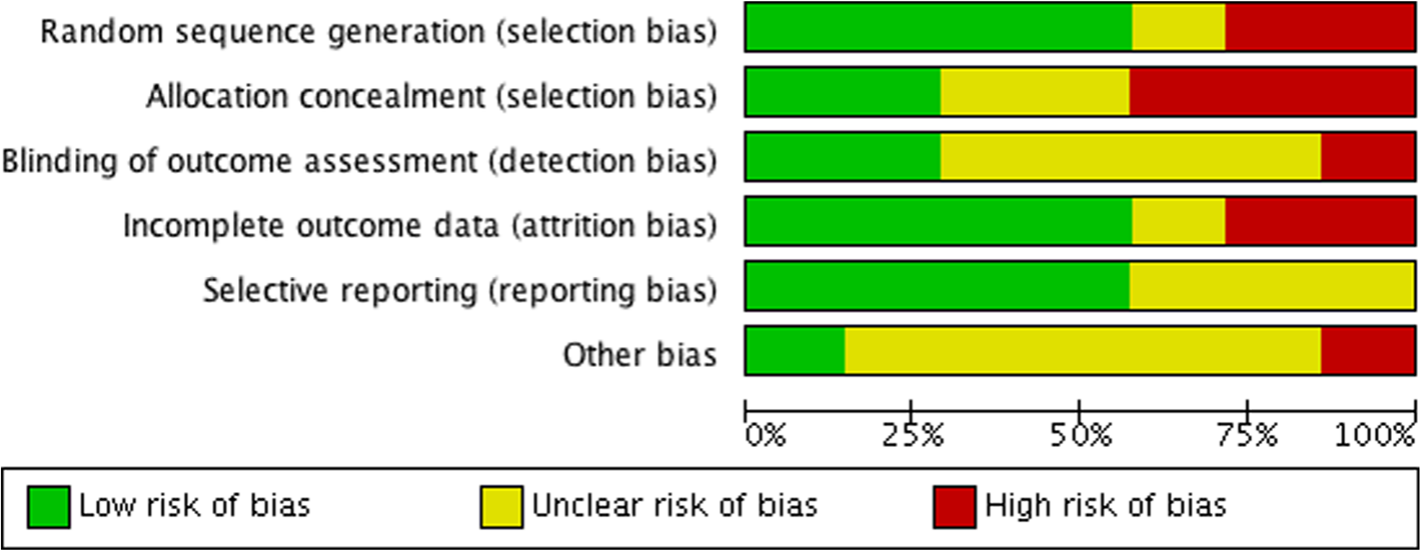
Graphic visualization of the risk of bias judgements

The review author’s judgment about each risk of bias item for each included RCT is presented in Table 3 and Fig. 2. From the studies included in the meta-analysis, two studies were assessed at low risk of bias [11, 49], three studies at moderate risk [1, 28, 50], and two at high risk of bias [4, 52]. Risk of bias was not judged for the studies included in the qualitative synthesis that either had no control group, employed indirect anchorage (see above, the “Study selection” section), had more than one test group, or lacked a non-implant control group [5, 9, 48, 54, 57].

**Table 3:**
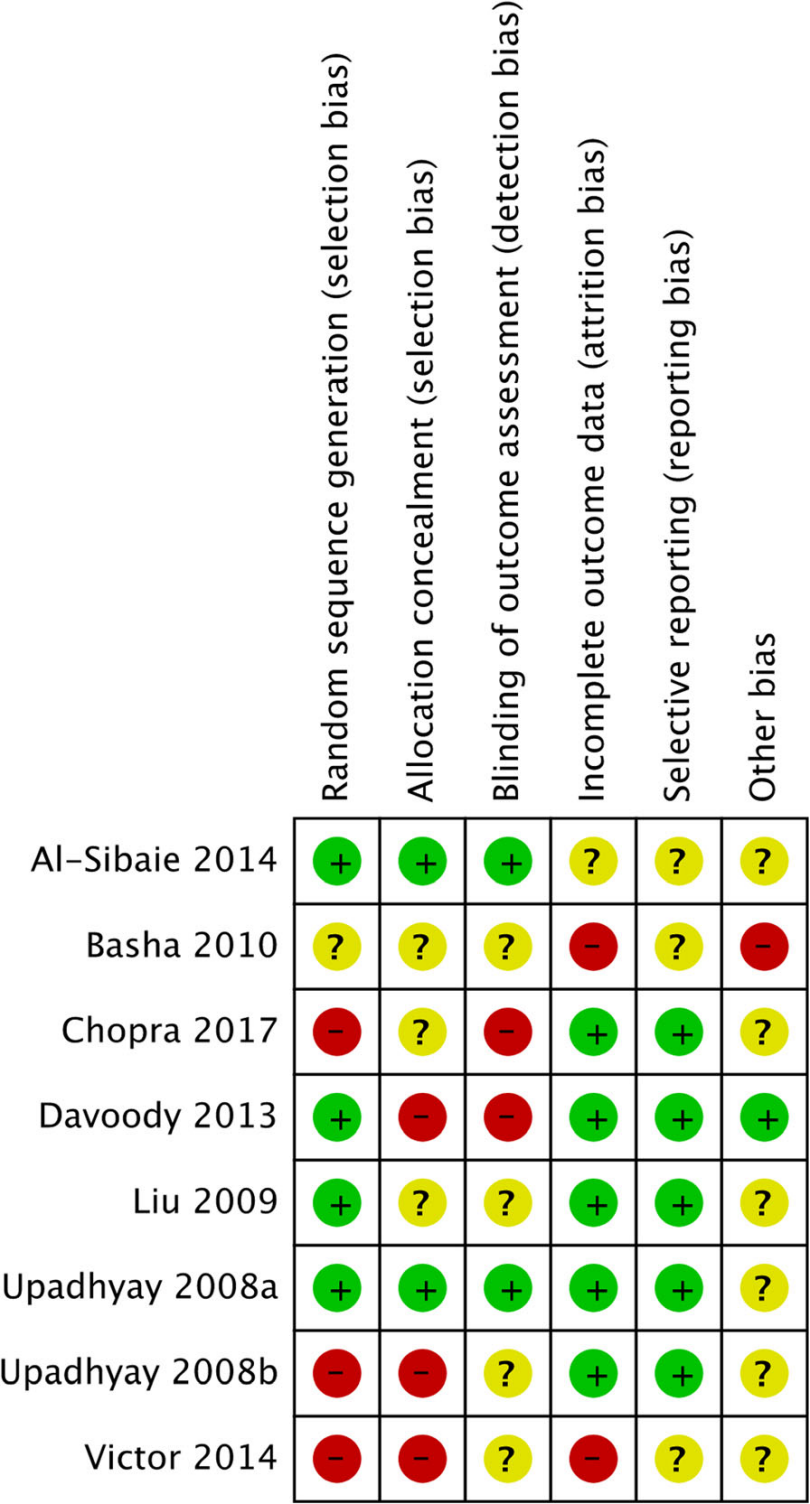
Risk of bias judgment according to the Cochrane Collaboration

#### Characteristics of the patients

The study samples consisted of patients exhibiting an Angle Class II,1 malocclusion [1, 5], patients with an Angle Class I requiring front retraction with maximum anchorage [4], patients exhibiting dental bi-maxillary protrusion with Angle Class I [9], patients with a need for extraction of four premolars (one in each quadrant) and maximum anchorage for front retraction [28], patients in need of extraction of the first upper premolars and front retraction [52], Angle Class I [49], patients with Angle Class II,1 with dental protrusion [50], or either Angle Class I or Class II with dental protrusion [11].

The study samples considered for the qualitative synthesis consisted of females exhibiting Angle Class II,1 malocclusion with upper dental protrusion and an overjet of at least 7 mm [48], patients with a dental Class II, a need for extraction of the first upper premolars and front retraction [54], or Class III patients with a need for pre-surgical decompensation through premolar extraction and front retraction [57].

#### Interventions

The majority of studies employed mini implants in direct anchorage mode placed bilaterally in the alveolar ridge. After leveling, alignment, and placement of a passive stainless-steel arch (varying from 0.019″ × 0.025″ to 0.016″ × 0.0022″), the implants were placed between the tooth roots. Retraction was achieved through sliding mechanics using either power chains or nickel titanium coil springs of usually 100–200 g. Implant lengths varied from 7 to 9 mm, and the diameter varied from 1.2 to 2.0 mm (Table 2). All implants were loaded within 3 days [1, 11, 28, 48–50, 52].

In the majority of the indirect anchorage groups, a single mini implant was placed in the anterior palate and connected to the first molars through an individually fabricated transpalatal arch [5, 54, 57]. Whereas three studies used the Straumann® Ortho (Basel, Switzerland) system and employed loading after 3 months of healing [5, 54], one study used either a 2 × 10 mm Dual Top™ (Jeil Medical Corporation, Seoul, South Korea) or a 2.0 × 11 mm BENEFIT® (Mondeal Medical Systems, Mühlheim, Germany) implant and employed immediate loading. One study employed indirect anchorage through a mini implant located in the alveolar ridge [9] (Table 2).

In the control groups, the majority of studies employed transpalatal arches. Interventions such as headgear, Nance button, intrusion arches, and differential moments were also employed (Table 2).

### Effects of intervention

#### Anchorage loss

Anchorage loss was a common finding for all control interventions. In the test groups, anchorage loss was also associated with indirect anchorage using mid-palatal implants. Mesial tooth migration was always lower in indirect anchorage mode compared to conventional anchorage groups (if evaluated) [5, 54, 57].

In detail, anchorage loss associated with indirect anchorage and a mid-palatal implant amounted to 1.5 ± 2.6 mm versus 3 ± 3.4 mm [5], 0.7 ± 0.4 (right molar) and 1.1 ± 0.3 mm (left molar) [54], 1.73 ± 0.39 mm (horseshoe), and 0.36 ± 0.11 mm (posterior reinforcement) versus 4.21 ± 1.17 mm [57]. An anchorage loss of 0.2 ± 0.35 mm versus 2.0 mm ± 0.65 mm was also observed in one study employing indirect anchorage using two implants in the alveolar ridge [9].

In contrast, no anchorage loss [4] or anchorage gain/reverse anchorage loss (distal movement) was observed in the groups employing *direct* anchorage through implants located interdentally in the alveolar ridge [1, 11, 28, 48–50, 52].

Vertical anchorage loss with molar extrusion was another common observation for the control interventions [1, 11, 28, 49, 50, 52]. In the majority of the studies, molar intrusion was commonly associated with direct skeletal anchorage [11, 28, 48–50, 52], but one study observed a minor extrusion tendency of 0.02 ± 0.93 mm associated with direct anchorage [1]. Vertical anchorage loss associated with indirect anchorage has not been evaluated.

Transversal anchorage loss with a mean expansion of 1.73 ± 0.39 mm following retraction was observed in one study employing indirect anchorage through a mid-palatal mini implant coupled with a horseshoe arch [57]. This tendency of transversal expansion could be reduced to 0.36 ± 0.11 mm by integration of a posterior reinforcement element. In contrast, a significant decrease in inter-molar width was observed in two studies employing direct anchorage through mini implants in the alveolar ridge [48, 50]. The inter-molar width reduction amounted to − 1.83 ± 1.29 mm [50] and may be counterbalanced by a transpalatal arch or by applying buccal crown torque on the molars [48]. The remaining studies, which analyzed lateral cephalograms only, did not report on anchorage loss in the transversal dimension. None of the studies compared transversal changes following skeletal anchorage with conventional control measures.

#### Retraction velocity and treatment duration

In the test groups, the monthly rate of posterior movement from the incisors amounted to 0.35 mm with a mean retraction duration of 12.9 months [1], 0.85 mm with a mean retraction duration of 6.0 months [4], 0.11 mm with a mean retraction duration of 21.76 months [9], 0.28 mm with a mean retraction duration of 26 months [28], 0.85 mm with a mean retraction duration of 8.61 months [49], and 0.44 mm with a mean retraction duration of 9.4 months [48].

#### Implant failures

The overall success rates of the orthodontic mini implants varied among the studies. A success rate of 95.7% with a loss of 2 from 46 implants was reported by Upadhyay et al. [48], and the implants could be replaced immediately. Two patients developed a peri-implant inflammation which was resolved through improved oral hygiene. A loss of 5 of 72 implants was reported by Upadhyay et al. [49], and in 2 patients, treatment was discontinued due to inflammation, which was resolved through improved oral hygiene. Davoody et al. [11] observed a success rate of 84% (5 of 30 implants), and Basha et al. [4] reported a success rate of 71.4%. In their study, 4 of 14 implants became loose during treatment but could be replaced subsequently. In further 4 patients, treatment was discontinued due to inflammation, which was resolved through improvement of oral hygiene. A success rate of 96% with a loss of 2 from 50 implants in the upper alveolar ridge due to peri-implant inflammation was observed by Chopra et al. [9], who employed indirect anchorage in the alveolar ridge. Similar values were reported by Benson et al. [5], who employed indirect anchorage through a mini implant in the mid-palate. In their study, in 6 of 24 patients, the implant failed to reach primary stability. In 4 patients, the implant had to be replaced during treatment, and in 2 patients, treatment was compromised due to implant failure. All implant failures occurred among the first implants placed by the surgeon, and no implant loss was observed for implants with sufficient primary stability.

A success rate of 100% with no signs of implant mobility, inflammation, or loss were observed in two studies [54, 57] in which indirect anchorage through mid-palatal implants was employed.

Summarizing these findings, implant loss was observed at 8 of 93 implants (8.6%) in the indirect anchorage group. In the direct anchorage groups, implant loss was reported for 16 of 162 implants (9.9%).

#### Meta-analysis

Meta-analysis was performed on RCTs reporting on anchorage loss at the first molar.

**Fig. 3:**
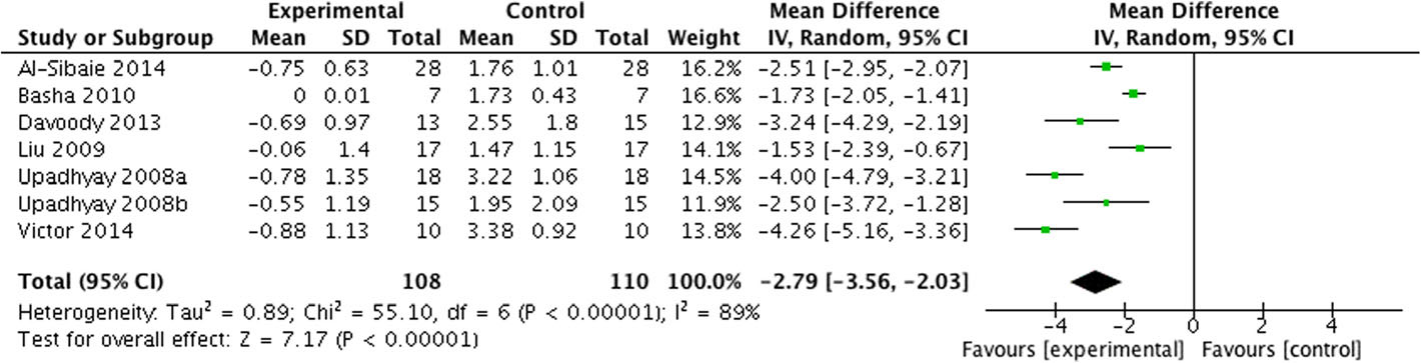
Forest plot for anchorage loss in the horizontal dimension

Based on seven studies [1, 4, 11, 28, 49, 50, 52], the weighted mean differences (WMD) [95% CI, *p*] in horizontal anchorage loss between test and control groups amounted up to − 2.79 mm [− 3.56 to − 2.03 mm, *p* < 0.0001] favoring skeletal anchorage over conventional anchorage devices (Fig. 3). The heterogeneity among the analyzed studies was high (*τ*^2^ = 0.89, *I*^2^ = 89%).

**Fig. 4:**
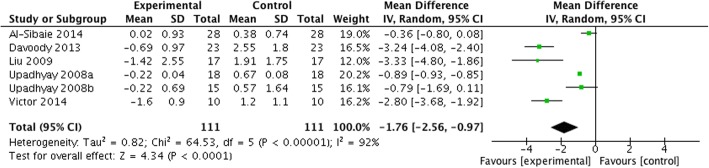
Forest plot for anchorage loss in the vertical dimension

Based on six studies [1, 11, 28, 48–50, 52], the WMD [95% CI, *p*] in vertical anchorage loss between test and control groups amounted to − 1.76 [−.56 to − 0.97 mm, *p* < 0.0001] favoring skeletal anchorage over control measures. The heterogeneity among the studies was high (*τ*^2^ = 0.82, *I*^2^ = 92%) (Fig. 4).

**Fig. 5:**
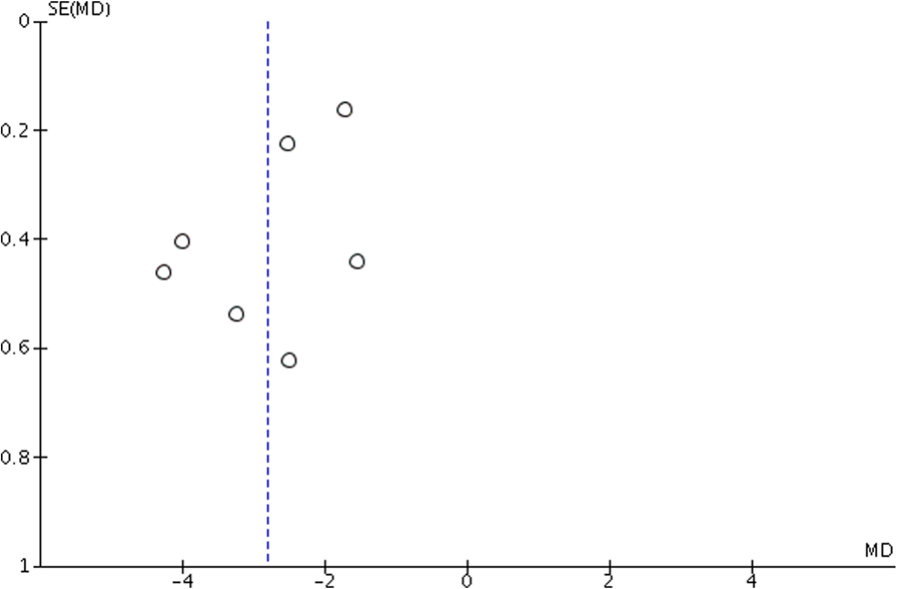
Funnel plot for anchorage loss in the horizontal dimension (MD mean difference, SE standard error)

**Fig. 6:**
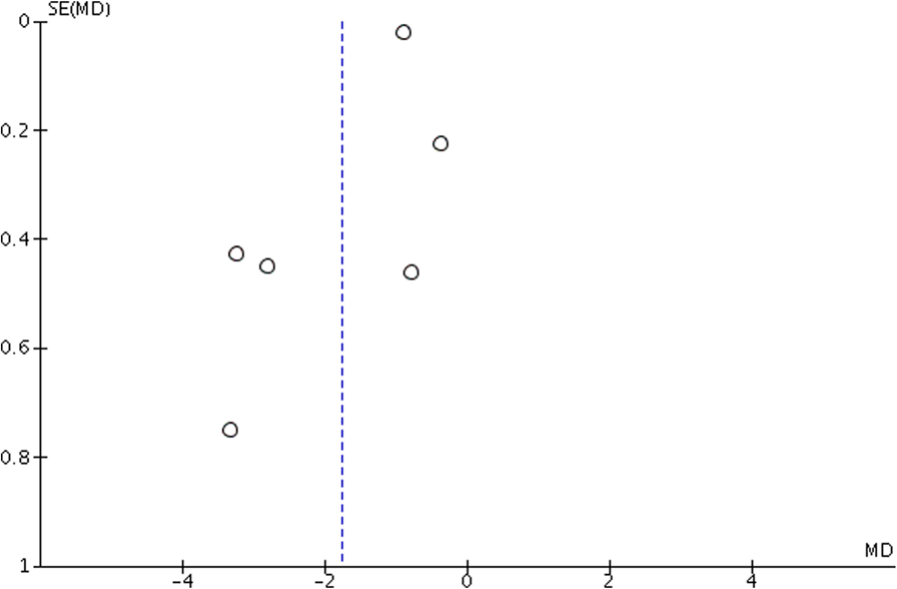
Funnel plot for anchorage loss in the vertical dimension (MD mean difference, SE standard error)

Funnel plots of the intervention effect estimates (presented as mean differences) plotted against standard errors are presented in Figs. 5 and 6. Their symmetricity suggests the absence of publication bias.

## Discussion

The present systematic review was conducted to address the following focused question: “In patients with a need for en masse retraction of the upper front teeth, what is the efficacy of orthodontic mini implants for anchorage control compared with conventional anchorage devices?”

The literature search revealed that efficacy of anchorage control of orthodontic mini implants in comparison to conventional devices was evaluated in nine randomized clinical trials (RCTs) [1, 4, 5, 9, 11, 28, 48–50, 52]. Seven of these studies employed direct anchorage in the alveolar ridge, whereas one study employed indirect anchorage together with a buccal implant [9], and one study used a mid-palatal implant and indirect anchorage [5]. Each of these studies reported on anchorage loss in the horizontal dimension, whereas vertical and transversal anchorage loss was only addressed in six and one of these studies, respectively. One cohort study also evaluated vertical anchorage loss associated with mini implants [48], whereas transversal changes have also been addressed in one controlled clinical trial and in one cohort study [54, 57].

Data syntheses of respective RCTs revealed a gain of anchorage for direct anchorage in the horizontal and vertical dimension, whereas indirect anchorage was associated with minor amounts of anchorage loss. Conventional treatments were commonly associated with a mesial migration and extrusion of the first upper molars.

Even though all studies favored orthodontic mini implants over conventional devices, distal migration and slight molar intrusion were only observed in groups employing direct anchorage through mini implants in the alveolar ridge. It has been suggested that the distal and intrusive forces result from the direction of the retraction forces causing some binding (or increase in friction) of the archwire to the brackets or tubes. Friction may have prevented sliding thus causing the force to be transmitted through the archwire to the dentition [11, 48, 50]. Whether this effect will be more pronounced if a coil spring is left in place for a couple of months after completion of front retraction as suggested by Upadhyay et al. [48] has not been analyzed so far. Notably, the observed effects varied from absolute anchorage with no tooth migration [4] to varying amounts of distal migration up to − 0.88 mm ± 1.13 mm. Hence, the underlying biomechanical causes need to be further analyzed.

Indirect anchorage through implants in the alveolar ridge was associated with mesial molar migration in all studies included in the present review [5, 9, 54, 57]. Nonetheless, anchorage loss with indirect anchorage was significantly lower compared to the conventional devices [5, 9, 57]. It has been suggested that the anchorage loss at indirectly anchored mid-palatal implants may be caused by a slight bending of the transpalatal bars which pass from the implant to the anchor teeth [54]. Additionally, implant migration, which describes a displacement of an implant while maintaining stability, may have contributed to the findings [5].

Transversal changes have not been compared to conventional devices, and controversial transversal effects have been reported in orthodontic mini implant groups [48, 54, 57]. Whereas an expansion tendency was observed in conjunction with palatal implants and indirect anchorage [57], inter-molar width reduction and palatal tipping of the molar crowns were observed in a study employing direct anchorage and implants in the alveolar ridge [48]. Hence, posterior reinforcement and application of differential moments have been suggested to avoid these side effects in the respective studies.

Implant loosening or complete failures have been reported in some studies, whereas others observed a 100% success rate. Discontinuation of treatment owing to inflammation was reported for implants placed in the alveolar ridge only. However, in several cases, resolution was successfully achieved through improved oral hygiene [4, 48, 49]. Whereas adverse effects including root damage, or loss of tooth sensibility have been reported in literature [15, 21], none of these complications were reported in the included studies. Also, no failures due to root contact have been reported in the included studies, even though root proximity is considered to be a major risk factor for implant loosening [53].

The implant failure rates of 9.9% and 8.6% were comparable between direct and indirect anchorage groups and also lower compared to the failure rate of 13.5% reported by two systematic reviews [2, 37]. Interestingly, two of three studies reporting on implant failures in the alveolar palate observed a 100% success rate that relates to the achievement of the respective treatment goal [54, 57]. In the other study evaluating mid-palatal implants, implant failure was observed only among the first series of implants placed by an unexperienced surgeon, and no implant losses were noted for implants that had reached primary stability [5]. This finding is in line with other studies reporting on high success rates for orthodontic implants in the alveolar palate [20, 24, 36, 58].

## Conclusions

The present systematic review and meta-analysis revealed that orthodontic mini implants are associated with a significantly lower anchorage loss at the first upper molars compared to conventional anchorage devices for en-masse retraction in the maxilla. However, the ideal implant location (anterior palate versus alveolar ridge) and the most beneficial concept (direct or indirect anchorage) need to be further evaluated. The heterogeneity was high among the included studies, control groups were not always homogenous, and two included studies were judged of high risk of bias. Further high-quality prospective, randomized clinical trials are needed to investigate the anchorage efficacy of orthodontic mini implants in comparison to conventional techniques.

## Additional file

Additional file 1: Data extraction template. (CSV 2 kb)
